# Ecological and socioeconomic factors associated with globally reported tick-borne viruses

**DOI:** 10.1038/s43856-026-01461-5

**Published:** 2026-03-02

**Authors:** Samantha Sambado, Sadie J. Ryan

**Affiliations:** 1 Department of Ecology, Evolution and Marine Biology, University of California, Santa Barbara, Santa Barbara, CA USA; 2 Department of Biology, Stanford University, Stanford, CA USA; 3 Quantitative Disease Ecology and Conservation (QDEC) Lab, Department of Geography, University of Florida, Gainesville, FL USA; 4 Emerging Pathogens Institute, University of Florida, Gainesville, FL USA

**Keywords:** Viral infection, Databases

## Abstract

**Background:**

Public health resources are often allocated based on reported disease cases. However, for under-recognized infectious diseases such as tick-borne viruses, risk assessments should also account for ecological and socioeconomic factors that influence disease case reporting. This study identifies country-level predictors of tick-borne virus reporting and evaluates whether wealthier nations are more likely to report resource-intensive cases.

**Methods:**

We applied boosted regression trees, a robust machine learning algorithm, to a comprehensive global database of tick-borne viruses and 24 environmental and socioeconomic variables.

**Results:**

Countries with lower income inequality and greater expertise in verterinary, agricultural, or forestry sectors are more likely to report tick-borne virus cases. Wealthier nations with stronger institutional and professional capacity exhibit higher reporting rates, whereas countries affected by conflict or limited health infrastructure show underreporting. Climatic factors, particularly subartic environments, also contribute to reporting likelihood, complementing the effects of socioeconomic drivers.

**Conclusions:**

Disease reporting is shaped by both ecological context and socioeconomic capacity. Strengthening surveillance through targeted resource allocation and better integration of veterinary and public health expertise under the One Health framework could enhance global tick-borne disease mitigation. These findings provide valuable evidence to support the World Health Organization’s Global Arbovirus Initiative and emphasize the need for equitable disease surveillance across regions.

## Plain language summary

Public health resources are often allocated based on reported disease cases, but for lesser-known diseases like tick-borne viruses, reported cases may not match areas where the risk is highest. Detecting and reporting these diseases requires both individual awareness and institutional support, which may be limited in under-resourced countries. We examine how environmental and socioeconomic factors influence where tick-borne viruses are reported worldwide. Using regression-based machine learning algorithms, we find that both ecological and socioeconomic factors matter, and wealthier nations are more likely to report overlooked diseases. Integrating veterinary and public health expertise under a One Health approach, that recognizes the interconnectedness of human, animal, and environmental health, will strengthen disease surveillance and promote healthier communities.

## Abstract (web summary)

Sambado et al. examine how environmental and social conditions shape where tick-borne viruses are reported worldwide. Using machine learning, they find that both ecological and economic are important, and that wealthier nations are more likely to report these overlooked diseases.

## Introduction

Infectious disease occurs when people and pathogens align in space and time^1–3^. However, reporting an occurrence of an infectious disease involves more than just the biological aspects of the disease system^4–7^. From the initial infectious contact to the reporting of a disease case, several steps must be completed. First, individuals need to be aware of and recognize the disease symptoms. If health care is accessible, they must have the resources to attain care. Following this, a systematic process must be in place to record and report the incidence, which is typically managed at the administrative level rather than by individuals. While the environmental suitability of a pathogen to persist in its natural cycles is undeniably important^8–10^, socioeconomic factors often play a significant role in determining which diseases are reported^4,5,11^. Reported case numbers, in turn, influence how public health resources are allocated.

The intensity of contact between people and pathogens are shaped by the characteristics of the surrounding landscape^6,10,12,13^. Risk of pathogen exposure can arise from various sources, including occupational hazards (e.g., individuals involved in agriculture or livestock management), residential environment (e.g., living near habitats conducive to pathogens like forests or animal aggregations), and recreational activities (e.g., walking through wooded areas)^4,14–17^. In contrast, while pathogen exposure might be lower in affluent urban environments, resources to manage potential exposures are typically more accessible compared to less urbanized communities^5,11,16,18^.

Infectious diseases impose a disproportionate health, and economic burden, on low-income countries^19–22^. The source of this disparity—whether due to biophysical features (such as increased biodiversity leading to higher pathogen diversity observed at lower latitudes) or reliance on natural resource-based activities (such as hunting, agriculture, or pastoralism for primary economic activity)—is not fully resolved for all diseases^5,12,19,20^. Nevertheless, key ecological insights offer a clearer understanding of certain diseases, especially those with well-documented life cycles and significant clinical impact, such as those with high morbidity and mortality^12,15,23^. For diseases with unclear transmission cycles or ambiguous symptoms, accurately assessing the true burden across all communities remains a significant challenge^15,18,24,25^.

Launched in 2022 by the World Health Organization, the Global Arbovirus Initiative aims to address the increasing risk of arbovirus epidemics, including tick-borne viruses (TBVs). Ticks transmit a broader diversity of pathogens, including protozoa, rickettsiae, spirochetes and viruses, than any other arthropod group, contributing significantly to global economic and health burdens through their impact on livestock production, companion animal health, and human disease^15,24,26^. Tick-borne viruses, which are considered zoonotic infectious diseases, are reported to be increasing and spreading into new geographic regions at rates comparable to other zoonotic threats^15,24,27–29^. This reported increase may be influenced by advances in molecular techniques and potential study biases, rather than rising transmission rates^15,28,30–32^. Confirming a TBV often requires a multi-tiered approach, including accurate tick identification, recognition of noticeable symptoms, and reliable diagnostic tests^14,15,27,29,32^. While some tick-borne diseases, such as Lyme disease, Crimean-Congo hemorrhagic fever, and Tick-borne encephalitis are tracked by global surveillance platforms like WHO Disease Outbreak News, ProMed-mail, World Animal Health Information System, and National Immunization Technical Advisory Groups, this coverage does not reflect the full range of tick-borne diseases to which both urban and rural populations may be exposed^24^. Additionally, the knowledge bases for reporting and surveillance are still patchily distributed across geographies and stakeholders such as public health, veterinary, agricultural, and entomological agencies, as well as research hubs^26,33^. The high resource and knowledge requirements for reporting TBVs contributes to a greater likelihood of their detection in affluent communities, where such resources are more readily available, rather than in areas with the highest environmental and occupational risk^4,5,7^.

Addressing the impact of socioeconomic factors on treatment access and the prioritization of reported cases in public health decisions presents a crucial opportunity to develop strategies that can significantly alleviate the burden of disease^15,27,29,32^. Effective strategies often include socio-ecological levers, which focus on interrupting environmental exposure and reducing vulnerability^3–5^. These approaches have been successful in managing other infectious diseases with environmental transmission, such as mosquito control measures like bednets and water treatments^24,27,34^. However, for vectors that are relatively long-lived and less understood, similar strategies may not be effective for ticks and may need to be tailored to specific tick-borne diseases^29,34^. Nonetheless, it is essential to implement education campaigns, including increasing the popularity of community science programs, that empower patients and physicians with knowledge about TBVs^33^. Resources for effective tick control should be strategically designed at the residential level while implementing a standardized system for reporting and notifying about tick-borne viruses at local, regional, or national levels^33^.

Our study examines the environmental and socioeconomic factors driving reported TBVs at the country-level, aiming to identify potential interventions for enhancing recognition and reducing disease burden. We compile a database of 24 hypothesized drivers for each country and integrate it with the currently most comprehensive tick-virus database (ZOVER) to train our machine learning algorithm, boosted regression trees (BRTs). Generalized BRTs are well-suited for high-dimensional data and can effectively determine the relative importance of each driver on reported virus infected ticks^35–37^. This approach addresses some of the challenges in understanding TBV risks, which have been previously constrained by limited data availability and inadequate statistical tools^15,29^. This paper aims (1) to identify the key country-level environmental and socioeconomic characteristics associated with reported tick-borne viruses, and (2) to examine whether countries with greater economic resources have a higher likelihood of reporting these resource intensive incidences. We find that non-ecological factors influence reporting, and that resource availability at the country level increases the likelihood of reporting tick-borne viruses. Our findings highlight opportunities for the One Health framework to better integrate health and veterinary expertise to strengthen disease surveillance.

## Methods

### Data curation

#### Reported tick-borne viruses

**Fig. 1:**
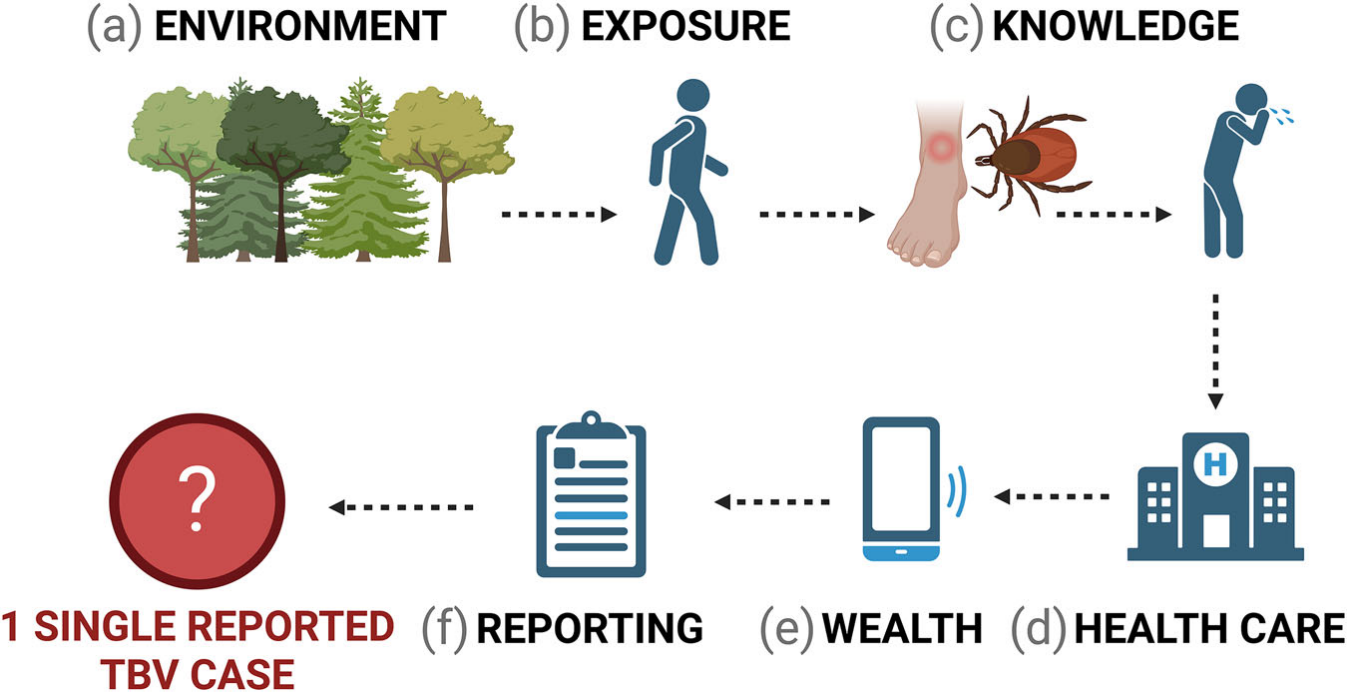
Illustration of the multi-tiered process involved in reporting a single tick-borne virus (TBV) case. It encompasses several critical factors. **a** Suitable environment for tick-borne pathogens; **b** human exposure to an infected tick; **c** awareness of ticks and associated disease symptoms (i.e., knowledge); **d** access to health care and **e** necessary resources for treatment (i.e., wealth); **f** a reporting system for documenting cases and estimating relative disease risk. Created in BioRender. Sambado, S (2025) https://BioRender.com/t28q173.

To assess our key focus (Fig. 1)—reported tick-borne viruses at the country level – we use data from the most comprehensive current database on zoonotic and vector-borne viruses (ZOVER, accessed on 21 09 2024). Data on ZOVER were manually gathered from published literature and records from the GenBank database^30,38^. While ZOVER data was reported as infected ticks, we assume that if a country is screening ticks for a specific virus, it likely has the resources to report human cases of the same virus. We compiled a total of 4213 unique records for the top tick-borne viruses of concern based on recent literature: African swine fever, Alkhumra hemorrhagic fever, Bhanja virus, Bourbon virus, Colorado tick fever virus, Crimean-Congo hemorrhagic fever, Deer tick virus, Heartland virus, Jingmen tick virus, Kyasanur forest disease virus, Louping ill virus, Lumpy skin disease, Nairobi sheep disease virus, Omsk hemorrhagic fever, Powassan virus, Sawgrass virus, Severe Fever with Thrombocytopenia Syndrome virus, Tick-borne encephalitis (Table 1)^14,24,28,31,39^. The top TBVs of concern represent seven virus families (*Asfarviridae*, *Flaviviridae*, *Nairoviridae*, *Orthomyxoviridae*, *Phenuiviridae*, *Poxviridae*, *Reoviridae*), two DNA viruses, and 12 RNA viruses (1 dsRNA, 11 ssRNA). Known tick vectors of these viruses include the genera *Amblyomma*, *Dermacentor*, *Haemaphysalis*, *Hyalomma*, *Ixodes*, *Ornithodoros*, *Rhipicephalus*. Human clinical symptoms from these viruses can be a mild, nonspecific febrile illness or can include severe, cognitive impairment^14,31^. Some of these viruses (Crimean-Congo hemorrhagic fever, Tick-borne encephalitis) are well documented, however many of these viruses have unknown enzootic cycles and limited knowledge of geographic risk^24,32^.

**Table 1:** List of viruses included in this study including disease name, disease abbreviation, virus family, known vectors of virus, and number of PubMed citations per disease (accessed on 09 08 2024)

Disease	Abbrev.	Viral family	Vector Genus	PubMed
African swine fever virus	ASFV	Asfarviridae	*Ornithodoros*	3253
Alkhumra hemorrhagic fever virus	AHFV	Flaviviridae	*Ornithodoros*	33
Bourbon virus	BRBV	Orthomyxo-viridae	*Amblyomma Haemaphysalis*	43
Colorado tick fever virus	CTFV	Spinareoviridae	*Dermacentor*	190
Crimean-Congo hemorrhagic fever virus	CCHFV	Nairoviridae	*Amblyomma* *Hyalomma* *Haemaphysalis* *Rhipicephalus*	1882
Deer tick virus	DTV	Flaviviridae	*Ixodes*	1590
Heartland virus	HRTV	Phenuiviridae	*Amblyomma*	111
Jingmen tick virus	JMTV	Flaviviridae	*Rhipicephalus* *Haemaphysalis* *Ixodes*	56
Kyasanur forest disease virus	KFD	Flaviviridae	*Haemaphysalis*	264
Lumpy skin disease virus	LSDV	Poxviridae	*Hyalomma* *Rhipicephalus*	576
Nairobi sheep disease virus	NSDV	Nairoviridae	*Haemaphysalis*	131
Powassan virus	POWV	Flaviviridae	*Ixodes* *Dermacentor* *Haemaphysalis*	4211
Severe fever with thrombocytopenia syndrome virus	SFTF	Phenuiviridae	*Haemaphysalis*	1431
Tick-borne encephalitis virus	TBEV	Flaviviridae	*Dermacentor* *Ixodes* *Haemaphysalis*	5501

Virus name and viral family come from NCBI Taxonomy Browser (https://www.ncbi.nlm.nih.gov/Taxonomy/Browser/wwwtax.cgi).

**Fig. 2:**
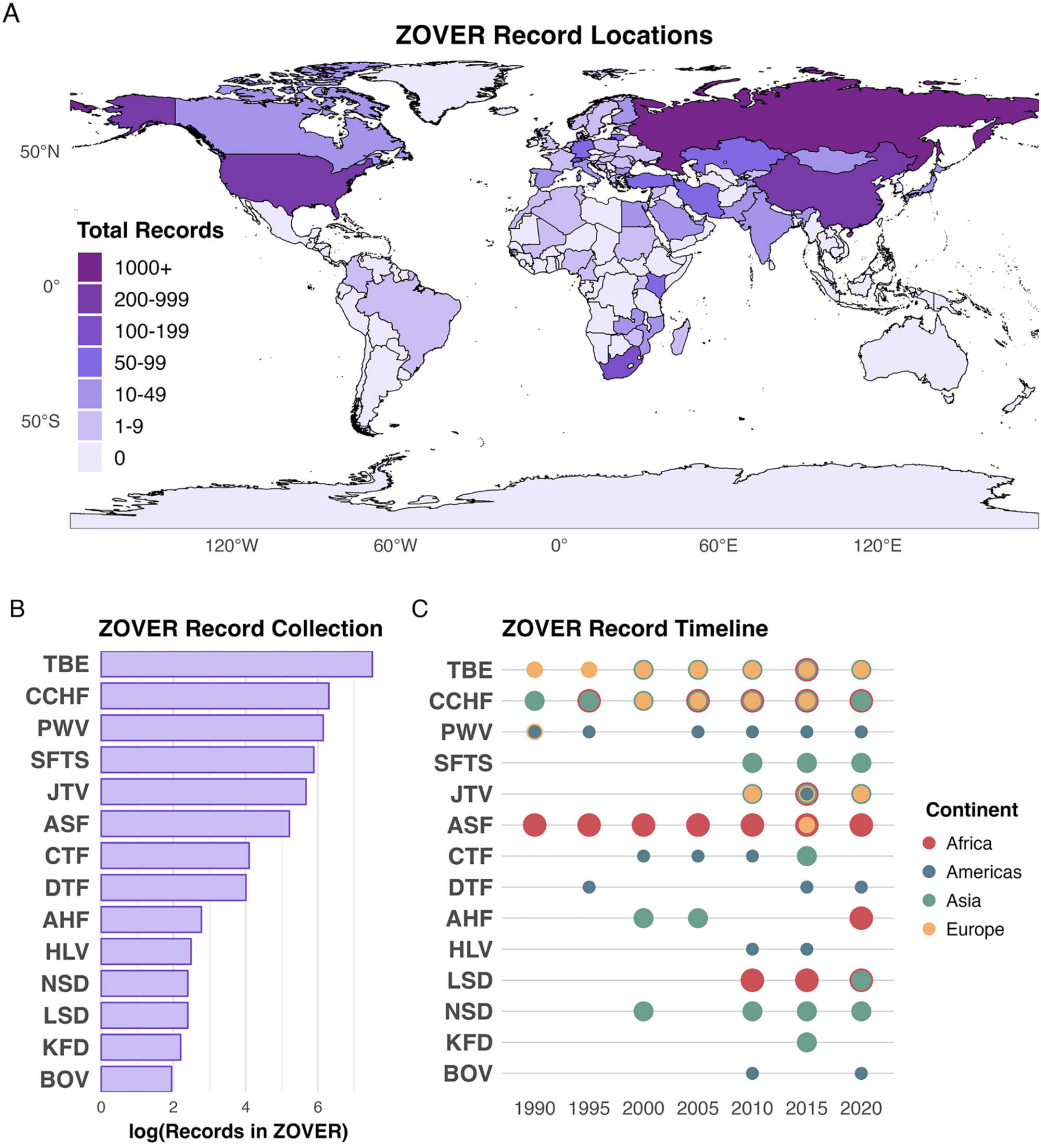
ZOVER database specifications used in this study. **A** Map of the top 14 reported tick-borne viruses for each county (more records – darker the color), **B** total number of records for each tick-borne virus, and **C** timeline of reported tick-borne viruses recorded every 5 years, color-coded and sized by the continent of origin (Abbreviated tick-borne virus names given in Table 1). The country boundary data are from the ‘rnaturalearth‘ package based on data from Natural Earth (naturalearthdata.com)^40^.

For our analyses, we restricted ZOVER records from the years 1990 to 2023 to match the temporal resolution of our covariates and reduce historic reporting biases, which reduced our observations to 3845 and eliminated observations for Bhanja virus, Louping ill virus, Omsk hemorrhagic fever virus, and Sawgrass virus (Fig. 2). Tick-borne virus names were cleaned and filtered for our top 14 reported viruses of concern, referred to as tick-borne virus (TBV) hereafter (see Supplementary text for more details on data cleaning). We began with the full country list from the package ‘rnaturaleath’ (*n* = 258 countries) and then excluded countries without reported square area in World Bank since most of them were small islands (*n* = 43)^40^. Each remaining country was assigned a binary code based on the presence (1) or absence (0) of a reported tick-virus in the ZOVER database (Supplementary Table 1). To address the additional bias introduced into the dataset, we obtain the number of citations per tick-borne virus species using the ‘easyPubMed’ package as a proxy for sampling effort^36,41^. We aggregate the citations for each tick-borne virus by country to capture the overall citation records and assess the relevance of each virus species (Supplementary Fig. 1).

#### Global trait matrix

The covariates selected for our analysis were informed by a conceptual framework that reflects multiple stages in the pathway from pathogen presence to formal disease reporting (Fig. 1). Each covariate represents a proxy for ecological and epidemiological factors known or hypothesized to influence TBV occurrence and reporting^4,5,15,39,42^. Environmental suitability variables capture broad-scale habitat suitability for ticks and their wildlife hosts, which is essential for the maintence of TBVs in enzootic cycles. Exposure risk factors represent the likelihood of human-tick interactions, which may be amplified by behaviors such as working or recreating in environmentally suitable areas. Knowledge capacity proxies indicate the population’s or institutions’s ability to understand local TBV risk and recognize disease symptoms, which can influence both care-seeking and diagnostic outcomes. Health care access reflects whether individuals can obtain medical attention once symptomatic, a necessary step before diagnosis or reporting can occur. Economic capacity (e.g., wealth indicators) reflects broader structural factors, such as the ability to invest in surveillance infrastructure or fund public health campaigns. Reporting structures reflect weather health systems are equipped to document and relay disease cases from individuals to centralized reporting databases, an essential final step in the disease reporting cascade. Together, these covariates aim to account for the ecological conditions that allow for TBVs to persist in nature and the societal factors that influence if an infection will go detected and officially reported.

For each country, when data was available (see Supplementary table 2 for covariate coverage), we extracted a set of 24 covariates which fall into six broad categories: *environmental* suitability for ticks, *exposure* risk to ticks, likelihood of *knowledge* on tick-borne diseases, access to *health care*, *wealth* to attain care, and availability of *reporting* structures (see Table 2). We prioritized covariates supported by peer-reviwed sources to improve the reproducibility and interpretability of our analysis. Covariates included the mode Köppen-Geiger Climate Classification within a country^43,44^, total number of ecoregions within a country^45^, mean vegetation heterogeneity per country^46^, % of total land area that is forested (FAO) (environment category); annual aggregate volume of livestock production (FAO), livestock density^47^, % of population employed in agriculture (ILO), (exposure category); pesticides use per capita (FAO), presence of an International Veterinary Students Association chapter (IVSA), % of graduates from tertiary education graduating from Agriculture, Forestry, Fisheries, and Veterinary programs (WB), % of graduates from tertiary eduation graduating from Health and Welfare programs (WB), adult literacy rate (UNESCO), education expenditures (% of GNI) (UNESCO) (knowledge category); health care expenditures (% of GDP) (UNESCO), reported average of annual people being treated for neglected tropical diseases (WHO) (health care category); social vulnerability index (SEDAC), % of population living below national poverty line, % of population with access to electricity, Gini Index (wealth category); conflict exposure to total events^48^, % of population exposed to conflict^48^, human population count (SEDAC), total area of country (FAO), % of population living in urban areas (WB) (reporting category) (Table 2, Supplementary Table 3). When possible, covariate data was averaged between 2010 and 2023 for each country, or the most recent year available was chosen. See Supplementary text for further details on data decisions.

**Table 2:** Descriptions of covariates used for the BRT analyses

Driver Type	Variable	Description
Environmental	Total number of ecoregions	Large areas defined by distinct natural communities, reflecting their original extent before significant land-use change
Environmental	Name of Köppen-Geiger climate classification	Climate groups are defined by the types of vegetation in a specific region
Environmental	Vegetation heterogeneity index	Second-order dissimilarity of Enhanced Vegetation Index among neighboring pixels. Proxy for deforestation.
Environmental	% forested land	% of total land area that is forested
Exposure	Livestock production index	An index measure of annual agricultural output relative to 2014–2016 base period
Exposure	Livestock density	Gridded global distribution of cattle expressed in total number of cattle per pixel
Exposure	% employed in agriculture	% of total employment involved in agriculture
Knowledge	Pesticide use	Pesticides use per capita (kilometers per person)
Knowledge	Presence of an International Veterinary Students Association (IVSA) chapter	IVSA aims to benefit animals and humans globally by leveraging the skills and dedication of veterinary students to promote veterinary education and practice
Knowledge	% of Ag, Vet, or Forestry graduates	% of graduates from tertiary education graduating from Agriculture, Forestry, Fisheries and Veterinary programmes, both sexes (%)
Knowledge	% of Healthcare or Welfare gradutes	% of graduates from tertiary education graduating from Healthcare or Welfare programmes, both sexes (%)
Knowledge	Adult literacy rate	% of people ages 15 and above literacy rate
Knowledge	Education expenditures	% of GNI spent on education expenditures (adjusted savings)
Health Care	Health care expenditures	% of GDP spent on healthcare expenditures
Health Care	NTD treatment	The number of people needing treatment for neglected tropical diseases (NTDs) targeted by the WHO NTD Roadmap and reported to WHO, including those requiring mass treatment for PC-NTDs (lymphatic filariasis, onchocerciasis, schistosomiasis, soil-transmitted helminthiases and trachoma).
Wealth	Gini index	Measure of income inequality within a population. Low values represent less inequality.
Wealth	% below poverty line	Poverty headcount ratio at national poverty lines (% of population)
Wealth	Social vulnerability index	A composite indicator, Subnational Human Development Index (SHDI), of relative multidimensional deprivation, based on a combination of three dimensions: education, health, and standard of living. Low values represent less vulnerability.
Wealth	% electricity access	% of population with access to electricity
Reporting	Human population counts	Gridded population counts
Reporting	Land area (sq km)	A country’s total area, excluding inland water bodies, continental shelf claims, and exclusive economic zones.
Reporting	Conflict exposure (total events)	Population that is living in an area of active disorder, where people may be directly injured, caught in conflict, targeted, or affected by the destruction of their community.
Reporting	Conflict exposure (% of population exposed)	‘Conflict exposure’ is a measure of the number of people living within 5 km of each conflict incident or demonstration.
Reporting	% urban population	% of total population living in urban areas

### Statistical analysis

We applied generalized boosted regression trees (BRTs), a machine learning algorithm, to identify environmental and socioeconomic characteristics at the country-level associated with (1) the presence or absence of a reported TBV and (2) citation effort, used as a proxy for sampling bias. Unlike traditional statistical models that fit a single parsimonious model, BRTs are an ensemble method that sequentially builds decision trees to improve model performance^35–37^. Through iterative “boosting”, each new decision tree is fitted to the residuals of the previous tree to increase emphasis on observations that were poorly predicted^35^. This approach allows BRTs to capture complex, nonlinear relationships among predictors without requiring strict assumptions about data distributions^35–37^. Outputs from the BRTs, such as variable importance values or partial dependence plots, can reveal general patterns in the data and inform the development of hypotheses for future research.

For this study, we developed two country-level BRT models: a classification model using a Bernoulli-distributed error to predict TBV presence or absence, and a regression model using a Poisson-distributed error to predict citation counts. The regression model was used to evaluate whether the predictors of TBV presence were primarily driven by underlying research effort rather than our hypothesized environment and socio-economic covariates. Prior to fitting the models, we removed covariates that had low data coverage (<50%) and minimal variation (>97% homogenous)^36,49^. Highly skewed variables (i.e., total area of country, human population counts, total citation counts) were log_10_-transformed to improve visualizations. Citation data were extracted using the ‘easyPubMed’ package and represented the total number of publications per virus per country^36,37,49^. We randomly divided the data into training (70%) and testing (30%) subsets using the ‘rsample’ package, applying stratified sampling to ensure equal representation of presence and absence observations^49,50^. To optimize model performance and avoid potential overfitting, we performed a grid search for hyperparamter tuning to determine optimal values for the number of trees, learning rate, and interaction depth^36,49^. The Poisson models used the same hyperparamters and covariates as the Bernoulli models^36,37,49^. All BRTs were fit using the ‘gbm’ package with ten-fold cross-validation and a bag fraction of 0.5^51^.

To determine model performance, we used different metrics for each model type. For the classification models, we assessed accuracy using the area under the receiver operator curve (AUC)^52^ and estimated sensitivity and specificity using the ‘InformationValue’ package^53^. For the regression models, we evaluated model performance using root mean squared error (RMSE) mean absolute error (MAE), and Spearman correlation ($\rho$) between predicted and observed citation counts. To account for variability due to random data partitioning, we repeated each model 100 times using stratified sampling and report mean performance metrics across all runs (e.g., $\overline{{AUC}}$ for accuracy; $\bar{X}$ for specificity and sensitivity)^49^. To identify the influence of each predictor on our TBV presence or absence outcome, we look at the relative variable importance, which indicates the proportion of improvement in model fit that is attributed to each predictor^51^. To visualize the marginal effects of an individual predictor variable on our outcome, while controlling for all other predictors, we created partial dependence plots^36,51^.

This study did not require ethical approval because it relied exclusively on publicly available, anonymized, and aggregated datasets. No new data involving human participants were collected or generated for the purposes of this research.

## Results

### Dataset description

A total of 59 countries have a reported TBV of top concern from the ZOVER database (Fig. 2, Supplementary Table 1). The country with the most reported unique TBVs was China (*n* = 6), followed by the United States (*n* = 5), and countries with at least three unique TBVs included Egypt, France, India, Japan, Kazakhstan, Kenya, Russia, and Serbia. However, the countries with the most recorded TBV observations were Russia (*n* = 1477), the United States (*n* = 580), and China (*n* = 278). The most reported TBVs were Tick-borne encephalitis (*n* = 1818), Crimean-Congo hemorrhagic fever (*n* = 546), and Powassan virus (*n* = 467). Most countries in our dataset only had 1 unique TBV (*n* = 44).

### Trait profile of countries with reported tick-borne viruses

**Fig. 3:**
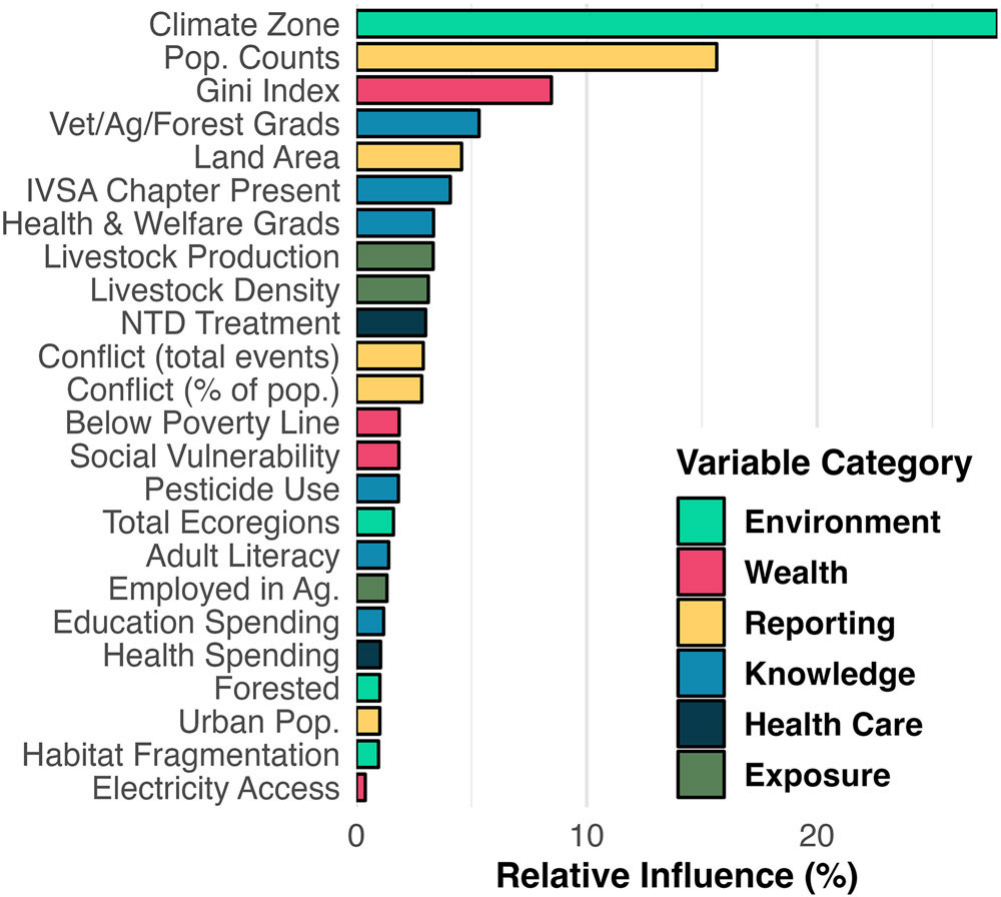
Variable importance values for the covariates in the BRT model. The value represents the proportion of improvement in model fit that is attributed to each predictor.

**Fig. 4:**
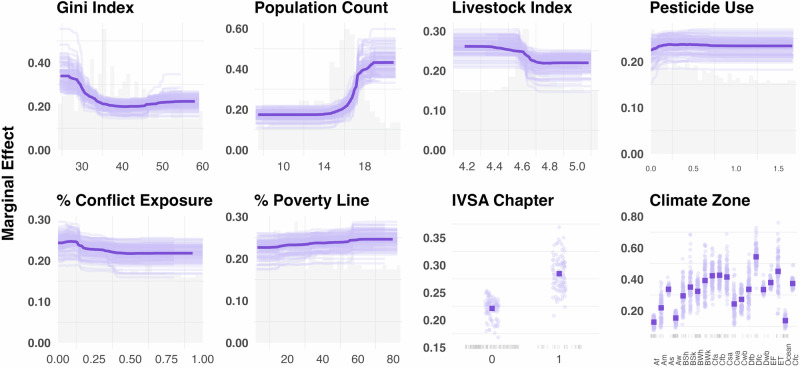
Partial dependence plots of some of the top BRT numeric predictors (Gini Index, log_10_-transformed human population counts, livestock production index, pesticide imports (USD$), % of population exposed to conflict, % of population living below the poverty line, presence of an International Veterinary Students Association chapter (IVSA, 1 = present; 0 = absent), mode Köppen-Geiger Climate Classification zone). Histograms and rug plots show the distribution of the underlying covariates. The thick purple line displays the average marginal effect of a given variable while controlling for all other predictors for predicting a country with a reported tick-virus. The thin purple line represents individual runs of our model.

To identify characteristics associated with tick-borne virus reported outcomes, we evaluated the relative importance of 24 variables with boosted regression tree (BRT) models. Our BRT model distinguished reported vs unreported cases with relatively modest accuracy ($\overline{{AUC}}$ = 0.83 ± 0.004) and specificity ($\bar{X}$= 0.90 ± 0.005), but low sensitivity ($\bar{X}$ = 0.43 ± 0.01). The mean relative influence ( ± variance) of all variables was 4.17 ± 35.64. The top three influential variables were Köeppen-Geiger climate classification zone (relative influence = 28%), human population count (relative influence = 16%), and Gini Index (relative influence = 8%) (Fig. 3). Other relatively important variables included the percentage of graduates from veterinary, forest, or agricultural programs, land area (sq km), the presence of an IVSA chapter, the percentage of graduates from healthcare and welfare programs, and livestock production index (Fig. 3, Supplementary Table 3). Partial dependence plots (Fig. 4, Supplementary Fig. 2) described countries that were more likely to report a TBV had a lower Gini Index (i.e., countries with less inequalities such as Nordic countries), increased pesticide use per capita, and had institutions (i.e., IVSA chapter) or individuals with agricultural, forestry, or veterinary knowledge (i.e., % of tertiary grads) present. Additional characteristics included countries with a lower percent of population exposed to conflict also had a higher probability of reporting a TBV. Among countries’ singular modal Köppen climate classifications, those with higher marginal contributions to TBV reporting included the continental subarctic climate (no dry season, cool summer) and the temperate oceanic climate (no dry season, warm summer), suggesting that environmental conditions characterized by these climate patterns may facilitate either increased exposure or better detection of TBVs. None of our variables had a relative influence of 0%, however the percentage of population with access to electricity had a relative influence of 0.38% (Supplementary Table 4). For our BRT models with a Bernoulli response (i.e., reported TBV), we identified an optimal learning rate of 0.0005, interaction depth of 3, and a maximum number of trees of 15,000 (Supplementary Fig. 3). To assess if our results from the Bernoulli model potentially reflected sampling biases, we ran a separate BRT model with citation count per country. This model showed that citation counts were only moderately predictable by our global trait matrix, with performance metrics of RMSE = 4.31 ± 0.02, MAE = 3.11 ± 0.02, and Spearman’s $\rho$ = 0.50 ± 0.01. These findings suggest that the predictors identified in our classification model are not soley reflecting citation effort but likely capture meaningful environmental and socio-economic factors associated with the presence of TBVs.

## Discussion

Public health resources and attention is allocated based on the relative magnitude of a number, specifically reported disease cases. However, reported incidence may not reflect true disease risk. Disease risk is a combination of environmental suitability for a pathogen to propagate and transmit as well as socioeconomic factors that influence the contact rates between pathogens and humans. Our study highlights the importance of both environmental and socioeconomic drivers of reported tick-borne viruses. Similar to other infectious diseases, both types of drivers are important to the transmission of infectious diseases^4,12^. However, our study highlights the inequities in reporting infectious diseases, particularly tick-borne viruses, which are more likely to be detected and documented in countries with greater financial resources, stronger public health infrastructure, and a workforce with relevant experience. Specifically, countries with higher GDP, a greater proportion of tertiary graduates in agriculture and veterinary science, and lower levels of conflict were more likely to report TBVs (Supplementary Fig. 2). These findings suggest that reporting is contingent not only on environmental suitability for pathogens, but also on access to diagnostic tools, trained professionals, and functioning surveillance systems, all of which are unevenly distributed globally. Due to the results of our study, we suggest socioeconomic interventions to be applied to countries with suspected TBVs to reduce the burden of tick-borne viruses globally. In accordance with other infectious diseases, by improving societal conditions, you can improve the health of all people^4,5^.

Environmentally mediated pathogens, such as tick-borne viruses that are vectored by free-living ectotherms, pose a significant global health burden^8^. However, unlike directly transmitted diseases (e.g., influenza, tuberculosis, measles), environmentally mediated pathogens allow for more interventions that can disrupt transmission to humans before requiring conventional medical approaches^4^. We find that countries are more likely to report a TBV if they were wealthy and had institutions (i.e., IVSA chapter) or individuals with agricultural, forestry, or veterinary knowledge (i.e., % of tertiary grads) present. Additional characteristics included countries with a lower percent of population exposed to conflict also had a higher probability of reporting a TBV. For a group of diseases that require specific knowledge and readily available diagnostic tools, our results make intuitive sense on a country-level^4,5,27^. If a country is unable to meet the basic needs of its citizens (i.e., high social vulnerability index, high percentage of population exposed to conflict, high percentage of population lives below the national poverty line) then it is unlikely to have resources for standardized surveillance to recognize and treat diseases that have less public health campaign efforts and less defined clinical symptoms^5,7,24^. Although much literature has supported that global disease burdens are concentrated in communities with less economic resources, environmental burden may not translate to reported disease cases^5,19,20^. Our work is supported by other country-level analysis of socio-economic-ecological drivers of environmentally mediated disease which found more affluent communities were more likely to report disease cases^4,5,7^. Potential mechanisms of this relationship include people in wealthier nations living in cities which may reduce distance to medical facilities and allow for a more efficient public health campaign^4,5,16,18^.

Although country-level analyses are often aligned with the scale most relevant for policy making, they come with important limitations^4,5^. Aggregating data at the national level can obscure finer-scale environmental heterogeneity, particularly climate patterns that are critical for the distribution and seasonality of exposure to TBVs^24^. To account for this, we included environmental covariates that represent suitable tick habitats such as Köppen-Geiger Climate Classification zones. Our model identified these classifications are relatively important predictors; specifically, subartic climates were associated with a higher probability of reporting a tick-virus. These findings are consistent with prior work suggesting that land cover identity may be even more informative than climate alone for predicting suitable tick habitat^42^. However, some of these relationships may have been skewed due to the use of a single (modal) classification zone per country (e.g., subarctic climate for Russia, Finland, Sweden), which likely oversimplifies within-country environmental variation (Supplementary Fig. 3). Our findings are also contained by the available information on the ZOVER database, which is subject to reporting biases (Supplementary Fig. 4). Several notable counties with known occurrences of tick-borne viruses, such as Mexico, Tanzania, and many central American countries, were absent from the ZOVER database. This emphasizes the need for improved reporting systems and expanded surveillance in ecologically suitable but underrepresented regions^33,42^. We also acknowledge that alternative proxy datasets could be suitable for similar analyses. However, we intentionally prioritized peer-reviewed and widely used data sources to maximize reproducibility and interpretability.

While we sought to address data-related biases as thoroughly as possible, our methodological approach introduces its own set of limitaitons. Specifically, some TBVs and geographic regions may be overrepresented due to disproportionate research interest or variation in national surveillance and reporting policies. To reduce the influence of this potential reporting bias, we modeled TBV occurrence as a binary presence or absence outcome and focus on TBVs with well-established etiology and transmission pathways. This approach helps to mitigate the effect of uneven sampling and reporting intensity across countries. To further test whether our results were driven by potenetial sampling bias, we fit a second BRT model using citation counts as a Poisson-distributed response and the same covariates and hyperparameters used in the primary model (Supplementary Fig. 1). Our citation model yielded moderate predictive performance, suggesting that the covariates included in our primary model did not significantly predict reporting effort. This result increases our confidence that the observed associations in our primary model reflect ecological and socioeconomic patterences related to TBV occurrence, rather than the results of uneven research or reporting intensity.

Tick-borne diseases are a function of a complex chain of events, spanning the remit of multiple fields of investigation and understanding. Thus, they are suited to One Health framed approaches to best capture their dynamics and occurrence, and to thereby inform mitigation or intervention^24,32,33^. Ticks and the pathogens they harbor can thrive in various environments, including natural communities (such as enzootic cycles without human hosts), peri-urban areas (where humans and companion animals coexist), and economic activities (like animal husbandry, agriculture, and forestry). Due to this wide network of exposure, physicians, veterinarians, and occupational health experts should work together to develop, maintain, and share tick surveillance to achieve the common goal of reducing human disease burden^32,55^. A One Health surveillance system could strengthen communication between these groups to improve tick identification, distribution of diagnostic tests, and implementation of tick-prevention strategies^28,56^. While the creation of a database like ZOVER is a positive step toward reducing tick-borne diseases, it is crucial to enhance our understanding of the real environmental risks posed by TBVs through robust active surveillance of ticks and their natural hosts.

## Conclusion

Launched in 2022 by the World Health Organization, the Global Arbovirus Initiative aims to address the increasing risk of arbovirus epidemics, including tick-borne viruses. The initiative focuses on monitoring disease risk, strengthening vector control, and building a coalition of partners. Our study identifies potential socio-ecological levers to improve the reporting of tick-borne viruses at a country level. We find countries that were more likely to report TBVs had a lower Gini Index (i.e., countries with less inequalities such as Nordic countries) and institutions (i.e., IVSA chapter) or individuals with agricultural, forestry, or veterinary knowledge (i.e., % of tertiary grads) present. Additional characteristics included countries with a lower percent of population exposed to conflict also had a higher probability of reporting a TBV. Although the environmental suitability for a pathogen to persist is important, our study suggests policy aimed at economic development may be the most effective tool to reduce tick-borne viruses.

## Supplementary information

Supplemental material

## Data Availability

Sources of data used in this study are publicly available and cited in the manuscript text. Data sources include: Food and Agriculture Organization of the United Nations (FAO), International Labour Organization (ILO), International Veterinary Student Association (IVSA, https://www.ivsa.org), World Bank (WB), World Health Organization (WHO), UN Educational, Scientific and Cultural Organization (UNESCO), and NASA’s Socioeconomic Data and Applications Center (SEDAC). Additional information on data sources, including the spatial and temporal resolution used in this manuscript, can be found in Supplementary Table 3 and Supplementary Table 5 to recreate the analysis and figures. Data for Fig. 2 comes from the ZOVER database (http://vca.mgc.ac.cn:4433/cgi-bin/ZOVER/main.cgi/) and are freely available under the CC BY-NC license version 4.0.

## Code availability

The data and code to re-create figures and analysis are hosted on GitHub (https://github.com/sbsambado/global_tbvs) and stored on a Dryad repository (10.5061/dryad.1g1jwsv9w)^54^.
