# Supplementary material for: Ecological and socioeconomic factors associated with globally reported tick-borne viruses
Source: Commun Med (Lond). 2026 Mar 2;6:210. doi: 10.1038/s43856-026-01461-5 (PMC13069038; doi:10.1038/s43856-026-01461-5)
Supplement: Supplementary file 1 — Supplemental material [file 43856_2026_1461_MOESM1_ESM.pdf]

SUPPLEMENTARY MATERIAL

Communications Medicine

Ecological and socioeconomic factors associated with globally reported tick-borne viruses

Samantha Sambado[1,2*] and Sadie J Ryan[3,4]

Affiliation of authors

[1]Department of Ecology, Evolution and Marine Biology, University of California Santa Barbara, Santa Barbara, California, United States of America

[2]Department of Biology, Stanford, California, United States of America

[3] Quantitative Disease Ecology and Conservation (QDEC) Lab, Department of Geography, University of Florida, Gainesville, Florida, United States of America

[4] Emerging Pathogens Institute, University of Florida, Gainesville, Florida, United States of America

***Corresponding author**: Samantha Sambado, PhD; sbsambado@ucsb.edu; University of California, Santa Barbara. Marine Science Institute, Bldg. 520. Santa Barbara, California 93106-6150.

Table of Contents

Supplementary text. Further details on data processing and model decisions
Supplementary figure 1. Bar graph of citation weight per country
Supplementary figure 2. Additional partial dependence plots
Supplementary figure 3. Performance of hyperparameters in the grid search
Supplementary figure 4. Global distribution of important covariates
Supplementary figure 5. Map of ZOVER coverage
Supplementary figure 6. Köppen-Geiger Climate classification partial dependence plots
Supplementary table 1. List of countries included in analysis
Supplementary table 2. Coverage of covariates included in models
Supplementary table 3. Additional covariate source information
Supplementary table 4. Variable importance rank
Supplementary table 5. Extended data availability statement

Supplementary text.

Tick-virus occurrence data . Tick-virus data came from the most comprehensive current database on tick-borne viruses (ZOVER; http://www.mgc.ac.cn/cgi-bin/ZOVER/main.cgi). On ZOVER, we went to the page for tick-associated viruses (http://www.mgc.ac.cn/cgi-bin/ZOVER/mainTable.cgi?db=tick). On the tick-associated viruses page, under 'Browse by virus', for each virus grouping (i.e., ssDNA viruses, dsRNA viruses, dsDNA viruses no RNA stage, unclassified viruses, ssRNA negative-strand viruses, and ssRNA positive-strand viruses, no DNA stage) we click Show 'All' entries, and 'Save this table' as xls files. Data was accessed on 2024-09-21. Data cleaning steps can be found in '2_DataCleaning_ZOVER.Rmd' on GitHub repository https://github.com/sbsambado/global_tbvs. Specifically, for the collection year, if the year was reported as Year 1 ~ Year 2, we chose the first year (Year 1). We then aggregate observations by five years, with the exception of observations before 1950. Further steps were taken to ensure correct tick virus names for the top 18 viruses using a series of string commands in RStudio (e.g., grepl(), str_detect(), case_when()). To filter for our top tick-borne viruses of concern the following strings were used to filter for observations in ZOVER: "African swine", "Alkhumra", "Bhanja", "Bourbon", "Colorado", "Crimean", "Deer tick", "Heart", "Jingmen", "Kyasanur", "Louping", "Lumpy", "Nairobi", "Omsk", "Powassan", "Saw", "Severe", and "Tick-borne enceph".

Global trait matrix data. To ensure that multiple data sources could easily be merged, much attention was given to country names for each data source. We based our final country names on the `rnaturalearth` package using the command ne_countries(scale = "large", type = "countries", returnclass = "sf"). This resulted in 258 countries including US Naval Base Guantanamo Bay, Southern Patagonian Ice Field, and other countries that were mainly small islands. However, all covariate sources were matched against the `naturalearth` country list and if there was no observation for a country an NA was assigned for that particular covariate. Data cleaning and processing steps can be found in '1_DataCleaning_TraitMatrix.Rmd' on GitHub repository https://github.com/sbsambado/global_tbvs. Specifically, data from World Bank (e.g., land area, Gini Index, adult literacy rate, below national poverty line, health expenditure) were selected from 2018 or the most recent year of observation. For the adult literacy rate, many countries were not reported but some assumptions were made about G20 countries having a 99.9% adult literacy rate to ensure this covariate could be included in boosted regression tree analysis. For covariate data that was derived from gridded remotely sensed data, data was extracted using the `terra` package within country polygons.

PubMed citation count. To assess potential sampling bias in the scientific literature for each tick-borne virus, we queried citation counts from PubMed using the `easyPubMed` package in R (v2_13). Virus names were taken from our boosted regression tree dataset and checked to match nomenclature from NCBI Taxonomy Browser as well as common usage in the literature (e.g., "African swine fever virus" was simplified to "African swine"). To enhance the relevance of

search results, each query was appended with the Boolean string "AND (virus OR viral)", and total citation counts were retrieved using the 'get_pubmed_ids()' function; the process was fully automated. While we did not filter by publication type (e.g., research articles vs reviews), we used this approach as a coarse, high-throughput proxy for sampling effort rather than a reflection of disease prevalence or actual case reporting.

Supplementary figure 1. Total citations were extracted using the `easyPubMed` package and were aggregated by (A) country and (B) tick-virus. Total citation counts were $\log_{10}$-transformed for visualization purposes. Abbreviations for country and virus names can be found in S Table 1 and Table 1, respectively.

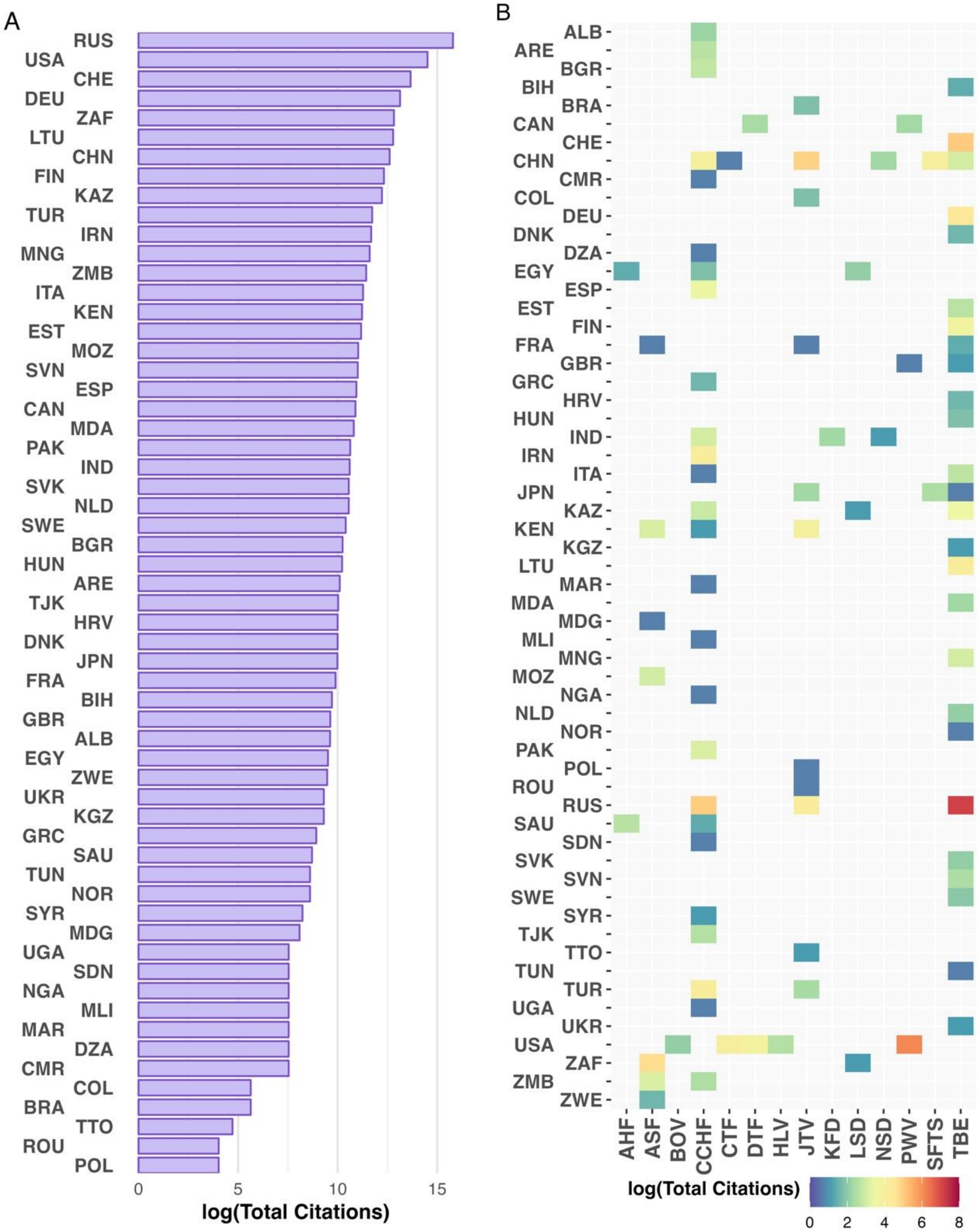

Supplementary figure 2. Additional partial dependence plots of BRT predictors for the Bernoulli distributed model (e.g., presence or absence of tick-borne virus). For non-numeric values (e.g., Climate Zone), the short vertical ticks along the x-axis indicate the total observations per grouping. For numeric values (e.g., Gini Index), histograms show the underlying data distribution. The thick purple line represents the mean marginal effect of a given variable for the prediction of tick-borne virus presence while controlling for all other predictors. Thin purple lines represent the outcome for individual model simulations.

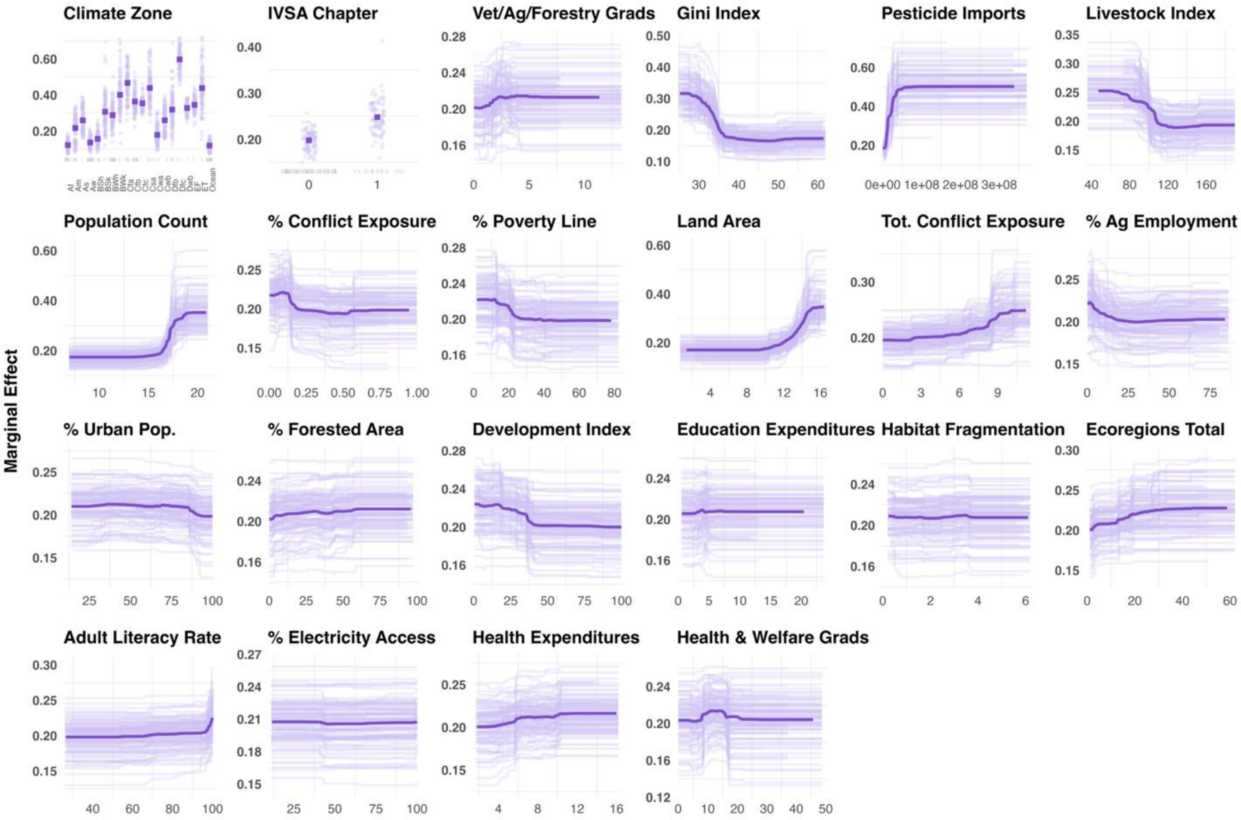

Supplementary figure 3. Results from a grid search for hyperparameter tuning of a boosted regression tree model. The search explored combinations of learning rate (i.e., shrinkage) and interaction depth (i.e., tree complexity), evaluating performance by test AUC. (A) Boxplots and jitter points show the distribution of test AUC across cross validation folds for each hyperparameter combination. (B) Heatmap of the mean test AUC for each combination of shrinkage and interaction depth. Models were trained with 5,000 trees.

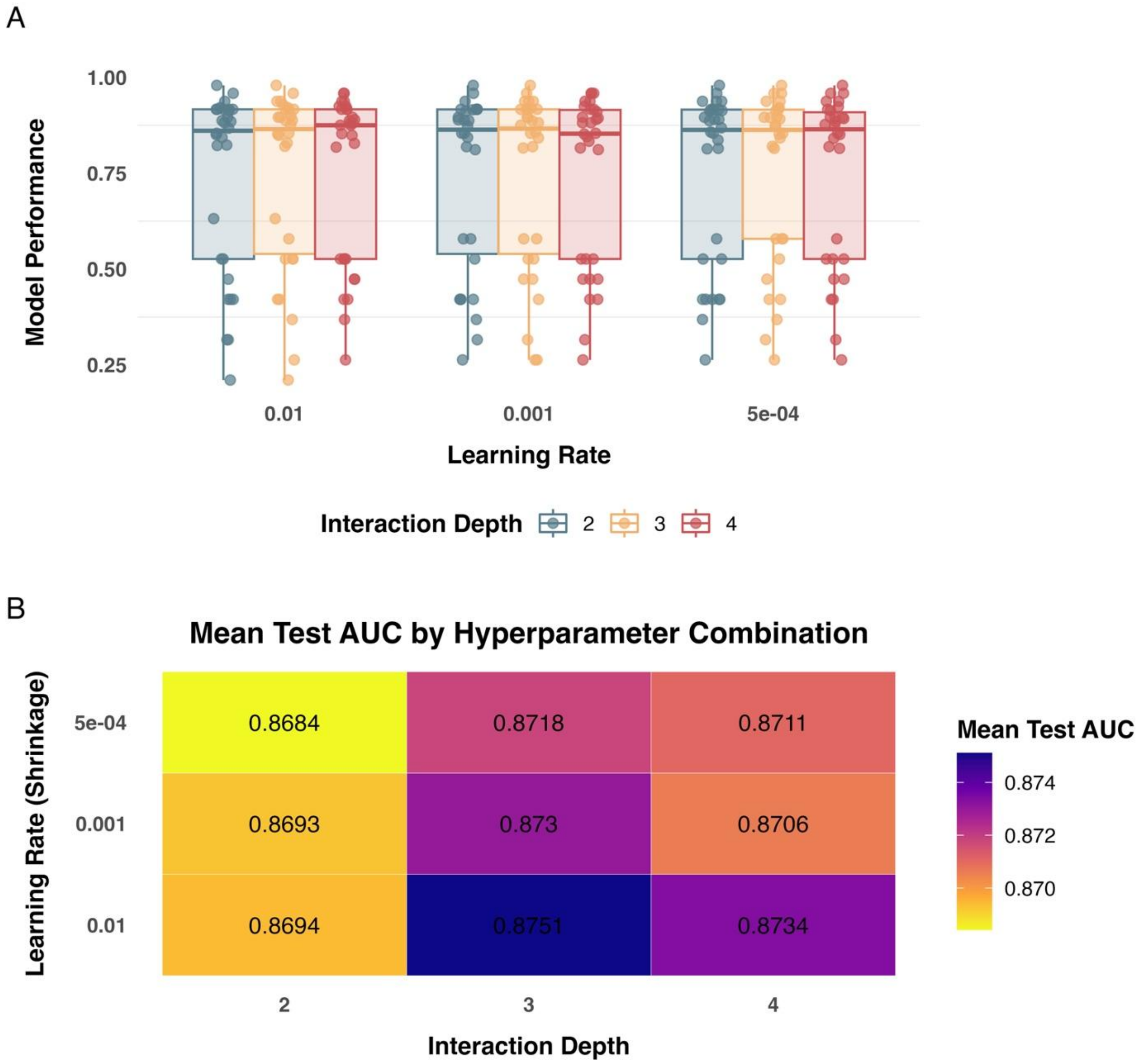

Supplementary figure 4. Global distribution of key predictors identified in the boosted regression tree analysis. Each map displays the mean national value of a covariate. Color intensity reflects the average value per country, with darker shades indicating higher values and lighter shades indicating lower values. National boundaries as defined by the `rnaturalearth` package.

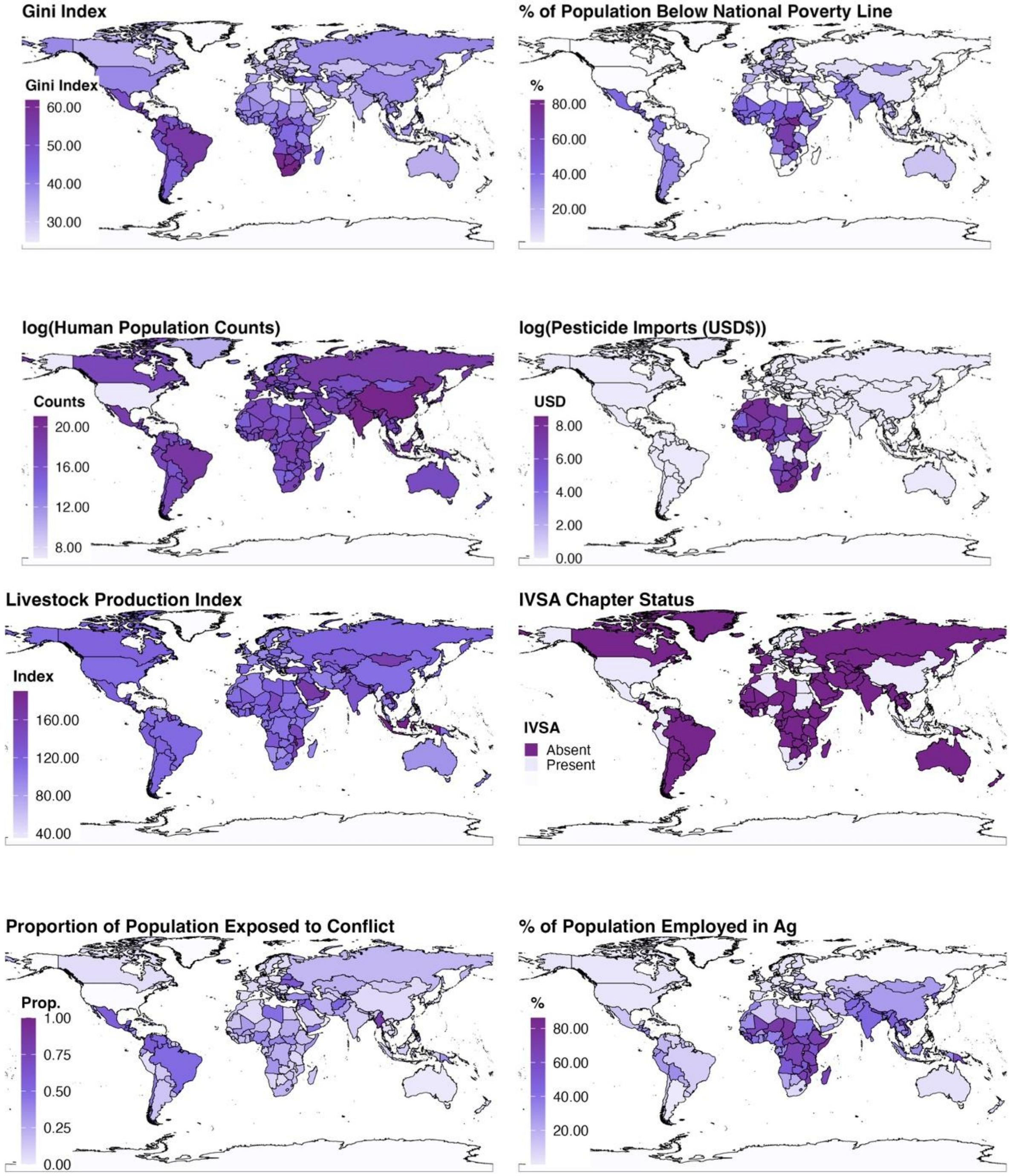

Supplementary figure 5. Global distribution of ZOVER coverage for tick-borne viruses (TBVs). (A) Coverage for selected TBVs reported between 1990 and 2023. (B) Coverage for all known TBVs reported between 1950 and 2023. Color intensity reflects the reported value for each country, with darker shades indicating higher values, lighter shades indicating lower values. National boundaries as defined by the `rnaturalearth` package.

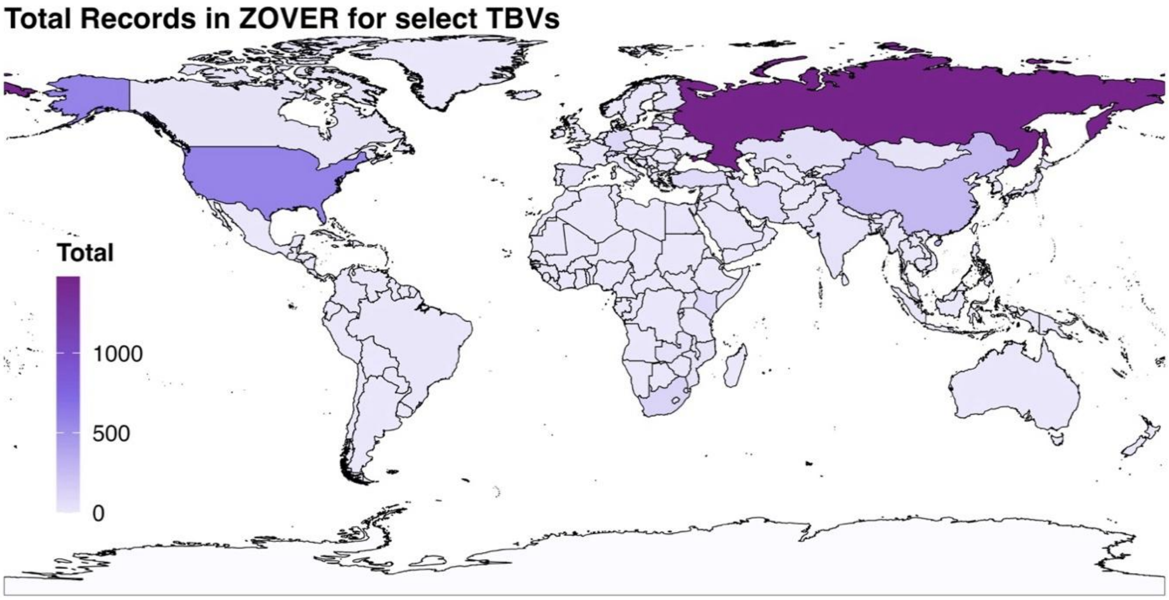

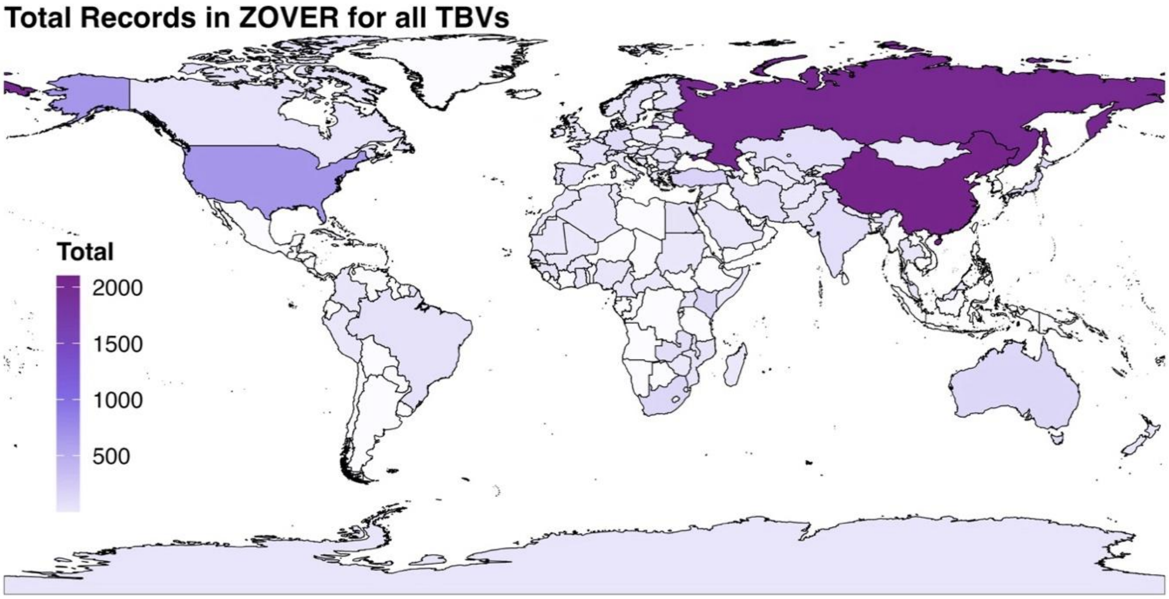

Supplementary figure 6. Partial dependence plot of predicted tick-borne virus (TBV) presence on Köppen-Geiger Climate classification zones from the boosted regression tree model. The plot shows the marginal effect of each climate zone on TBV presence while holding other variables constant. Points represent the average predicted contribution of each climate classification, reordered from most to least influential. Climate zone names come from Köppen climate classifications Wikipedia page (https://en.wikipedia.org/wiki/K%C3%B6ppen_climate_classification).

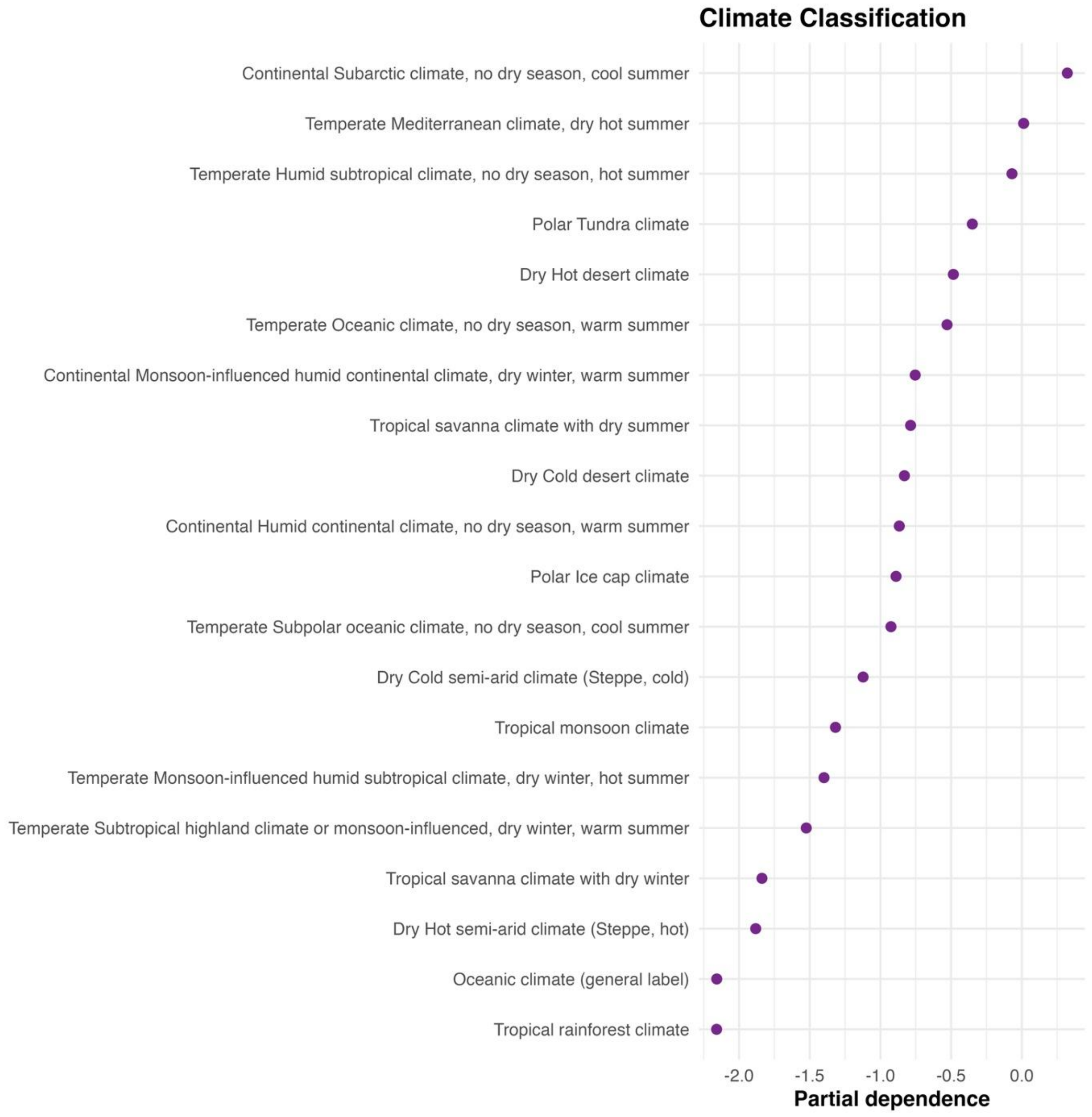

Supplementary table 1. List of TBVs by country. Tick-virus abbreviations: CCHF = Crimean-Congo hemorrhagic fever, TBE = Tick-borne encephalitis, JTV = Jingmen tick virus, DTF = Deer tick virus, PWV = Powassan virus, CTF = Colorado tick fever virus, NSD = Nairobi sheep disease virus, SFTS = Severe fever with thrombocytopenia syndrome virus, AHF = Alkhumara hemorrhagic fever virus, LSD = Lumpy skin disease virus, ASF = African swine fever virus, BHV = Bourbon virus, KFD = Kyasanur forest disease virus, HLV = Heartland virus.

Country	Total Reports	Reported Tick-borne Viruses
Russia	1477	CCHF, JTV, TBE
United States of America	580	BOV, CTF, DTF, HLV, PWV
China	278	CCHF, CTF, JTV, NSD, SFTS, TBE
Switzerland	156	TBE
South Africa	116	ASF, LSD
Germany	92	TBE
Turkey	76	CCHF, JTV
Kenya	73	ASF, CCHF, JTV
Lithuania	65	TBE
Iran	63	CCHF
Kazakhstan	50	CCHF, LSD, TBE
Finland	41	TBE
Zambia	33	ASF, CCHF
India	31	CCHF, KFD, NSD
Spain	30	CCHF
Pakistan	22	CCHF
Japan	21	JTV, SFTS, TBE
Mongolia	20	TBE
Canada	19	DTF, PWV
Mozambique	19	ASF
Saudi Arabia	16	AHF, CCHF
Bulgaria	15	CCHF
Egypt	15	AHF, CCHF, LSD
Italy	15	CCHF, TBE
Estonia	13	TBE
United Arab Emirates	13	CCHF
Tajikistan	12	CCHF
Slovenia	11	TBE
Moldova	9	TBE
Albania	8	CCHF
Netherlands	7	TBE

Slovakia	7	TBE
Sweden	6	TBE
Brazil	5	JTV
Colombia	5	JTV
France	5	ASF, JTV, TBE
Hungary	5	TBE
Croatia	4	TBE
Denmark	4	TBE
Greece	4	CCHF
Zimbabwe	4	ASF
Bosnia and Herzegovina	3	TBE
United Kingdom	3	PWV, TBE
Kyrgyzstan	2	TBE
Syria	2	CCHF
Trinidad and Tobago	2	JTV
Ukraine	2	TBE
Algeria	1	CCHF
Cameroon	1	CCHF
Madagascar	1	ASF
Mali	1	CCHF
Morocco	1	CCHF
Nigeria	1	CCHF
Norway	1	TBE
Poland	1	JTV
Romania	1	JTV
Sudan	1	CCHF
Tunisia	1	TBE
Uganda	1	CCHF

Supplementary table 2. Summary of feature variables used in the boosted regression tree models, including all numeric predictor variables and their corresponding short name used for visualization, data coverage (%), and data range.

Variable	Short name	Coverage	Range
TBV outcome	NA	100	0,1
log(Total citations)	NA	100	1-17
log(Land area (sq km))	Land area	100	1-17
% of population with access to electricity	Electricity access	99.5	6-100
% of population in urban areas	Urban pop.	99.5	13-100
log(human population Counts)	Pop. counts	99.1	7-21
% of land forested	Forested	99.1	0-95
Total ecoregions	NA	98.6	1-86
Social vulnerability	NA	97.2	0-100
Habitat fragmentation	NA	96.7	0-6
Education expenditures (% of GNI)	Education spending	93.0	0-24
log(Pesticide use per capita (km/person))	Pesticide use	91.2	0-2
log(Livestock density)	Livestock density	90.7	0-10
log (Livestock production index)	Livestock production	90.2	4-5
log(Annual people treated for NTDs)	NTD treatment	89.3	0-20
Healthcare expenditures (% of GDP)	Healthcare spending	89.3	2-19
% of population employed in Agriculture	Employed in Ag.	86.5	0-86
Conflict exposure (% of population exposed)	Conflict (% of pop.)	81.4	0-100
Conflict exposure (total events)	Conflict (total events)	81.4	0-12
% of population below national poverty line	Below poverty line	70.7	3-82
Gini index	NA	68.4	24-59
Adult literacy rate	Adult literacy	67.4	26-100
% of Tertiary Grad from Healthcare & Welfare	Healthcare/Welfare grads	60.9	0-53
% of Tertiary Grad from Ag, Vet, or Forestry	Vet/Ag/Forest grads	60.9	0-16

Supplementary table 3. Additional information on covariate sources, including the spatial and temporal scales of each variable. For gridded datasets (i.e., reported in kilometers), values were averaged within national boundaries as defined by the `rnaturalearth` package. Refer to Supplementary table 5 has a more comprehensive description of the variable are provided. *The average value of reported values during that time period.

Driver type	Variable	Spatial	Temporal
Environmental	Total number of ecoregions	Polygons totaled up per country	Based on the year 1995
Environmental	Name of Köppen-Geiger Climate classification	The mode polygons within country boundary was selected	Represents the conditions between 1986-2010
Environmental	Vegetation heterogeneity index	5 km	2005 (single time period)
Environmental	% of land forested	country	*2015-2023
Exposure	Livestock production index	1 km	2015 (single time period)
Exposure	Livestock density	country	*2015-2023
Exposure	% of population employed in agriculture	country	*2015-2023
Knowledge	Pesticide use per capita	country	2015-2017 (average)
Knowledge	Presence of an International Veterinary Students Association (IVSA) chapter	20 km	Based on presence in 2023
Knowledge	% of tertiary graduates from Ag, Vet, or Forestry programs	country	*2015-2023
Knowledge	% of tertiary graduates from Healthcare or Welfare programs	country	*2015-2023
Knowledge	Adult literacy Rate	country	*2015-2023

Knowledge	Education expenditures	country	*2015-2023
Health Care	Healthcare expenditures	country	*2015-2023
Health Care	Reported average of annual people being treated for NTDs	country	*2010-2023
Wealth	Gini index	5 km	*2015-2023
Wealth	% of population living below national poverty line	country	*2015-2023
Wealth	Social vulnerability index	20 km	*2010-2020
Wealth	% of population with access to electricity	country	*2015-2023
Reporting	Human population counts	5 km	2015
Reporting	Land area (sq km)	country	*2015-2023
Reporting	Conflict exposure (total events)	country	2020-2023 (summed)
Reporting	Conflict exposure (% of population exposed)	country	2020-2023 (summed)
Reporting	% of population in urban areas	country	*2015-2023

Supplementary table 4. Summary of variable relative influence across boosted regression tree models including the mean relative influence (%), standard error (SE), and variance (Var) for each predictor variable. Variables are ranked by their mean relative influence in descending order. The mean and standard error of relative influence across all variables were 4.17 and 1.22, respectively.

Rank	Variable	Relative influence	SE	Var
**1**	Köppen-Geiger climate zone	27.82	0.32	10.18
**2**	Human population counts	15.64	0.34	11.89
**3**	Gini index	8.47	0.29	8.72
**4**	% of grads from Ag/Vet/Forestry programs	5.33	0.22	4.78
**5**	Land area (sq km)	4.57	0.19	3.79
**6**	Presence of an IVSA chapter	4.08	0.23	5.46
**7**	% grads from Healthcare/Welfare programs	3.35	0.13	1.59
**8**	Livestock production index	3.34	0.13	1.80
**9**	Livestock density	3.13	0.13	1.60
**10**	Annual people treated for NTDs	3.01	0.11	1.21
**11**	Conflict exposure (total events)	2.91	0.12	1.41
**12**	Conflict exposure (% of population exposed)	2.84	0.15	2.15
**13**	% of population below national poverty line	1.86	0.06	0.32
**14**	Social vulnerability	1.85	0.12	1.49
**15**	Pesticide use per capita (km/person)	1.83	0.08	0.59
**16**	Total ecoregions	1.61	0.07	0.49
**17**	Adult literacy rate	1.40	0.07	0.48
**18**	% of population employed in agriculture	1.33	0.05	0.27
**19**	Education expenditures (% of GNI)	1.19	0.03	0.11
**20**	Healthcare expenditures (% of GDP)	1.05	0.04	0.17
**21**	% of land forested	1.02	0.04	0.14
**22**	% of population in urban areas	1.02	0.04	0.19
**23**	Habitat fragmentation	0.96	0.03	0.10
**24**	% of population with access to electricity	0.39	0.01	0.02

Supplementary table 5. Additional information on data availability. This table summarizes metadata for all variables used in the analysis. "Variable ID" corresponds to the term used in the manuscript figures. "Variable details" provide additional information to locate each indicator and its associated data source. The "Temporal scale" column describes the reference period or time range covered by each dataset. Many indicators were obtained from the World Bank (WB) using the `wbstats` R package (Piburn 2020 https://doi.org/10.11578/dc.20171025.1827). Data from the World Health Organization (WHO) were accessed via the `rgho` R package Filipovic-Pierucci 2024 https://CRAN.R-project.org/package=rgho). Full source attributions are listed below the table.

Source	Variable ID	Variable details
**1**	% of land forested	Forest area (% of land area)<br>(WB: AG.LND.FRST.ZS)
**2**	Livestock production index	Livestock production index (2014-2016 = 100)<br>(WB: AG.PRD.LVSK.XD)
**3**	Pesticide use per capita	Pesticides use per capita<br>(WB: FAO_RP_5172)
**4**	% grads from Vet/Ag/Forestry programs	Percentage of graduates from tertiary education graduating from Agriculture, Forestry, Fisheries and Veterinary programmes, both sexes (%)<br>(WB: SE.TER.GRAD.AG.ZS)
**5**	% of grads from Healthcare/Welfare programs	Percentage of graduates from tertiary education graduating from Health and Welfare programmes, both sexes (%)<br>(WB: SE.TER.GRAD.HL.ZS)
**6**	Adult literacy rate	Literacy rate, adult total (% of people ages 15 and above)<br>(WB: SE.ADT.LITR.ZS)
**7**	Education expenditures	Adjusted savings: education expenditure (% of GNI)<br>(WB: NY.ADJ.AEDU.GN.ZS)
**8**	Healthcare expenditures	Current health expenditure (% of GDP)<br>(WB: SH.XPD.CHEX.GD.ZS)
**9**	Gini index	Gini index<br>(WB: SI.POV.GINI)
**10**	% of population below national poverty line	Poverty headcount ratio at national poverty lines (% of population)<br>(WB: SI.POV.NAHC)
**11**	% of population with access to electricity	Access to electricity (% of population)<br>(WB: EG.EL.ACCS.ZS)
**12**	Land Area	Total Land area (sq. km)<br>(WB: AG.LND.TOTL.K2)

**13**	% of population in urban areas	Urban population (% of total population) (WB: SP.URB.TOTL.IN.ZS)
**14**	% of population employed in agriculture	Employment in agriculture (% of total employment) (modeled ILO estimate) (WB: SL.AGR.EMPLY.ZS)
**15**	Annual people treated for NTDs	Average annual number of people requiring mass treatment for at least one Neglected Tropical Disease (NTD) (WHO: SDGNTDTREATMENT)
**16**	Human population counts	SEDAC CIEN Gridded Population of the World (GPW), v4 (~5 km)
**17**	Social vulnerability	SEDAC CIEN Subnational Human Development Index (SHDI) from the Global Gridded Relative Deprivation Index (GRDI) (~20 km)
**18**	Livestock density	Gilbert et al. 2022. Livestock density (~1 km)
**19**	Habitat fragmentation	Tuanmu et al. 2015 Vegetation Dissimilarity (~5 km)
**20**	Total ecoregions	Olson et al. 2004 Ecoregions
**21**	Köppen-Geiger climate zone	Rubel and Kottek 2010 Koppen-Gieger climate classification zone
**22**	Conflict exposure (total events)	ACLED Total conflicts
**23**	Conflict exposure (% of population)	ACLED Percentage of population exposed to conflicts within 5 km
**24**	Presence of an IVSA chapter	IVSA Presence of an International Veterinary Student's Association chapter

Sources

[1]**World Bank.** "Forest area (% of land area)" *World Development Indicators*, Food and Agriculture Organization of the United States (FAO), https://data.worldbank.org/indicator/AG.LND.FRST.ZS. Accessed 31 Oct. 2025.

[2]**World Bank.** "Livestock production index (2014-2016)" *World Development Indicators*, Food and Agriculture Organization of the United States (FAO), https://data.worldbank.org/indicator/AG.PRD.LVSK.XD. Accessed 31 Oct. 2025.

[3]**World Bank.** "Pesticides use per capita" *World Development Indicators*, Food and Agriculture Organization of the United States (FAO), https://data360.worldbank.org/en/indicator/FAO_RP_5172. Accessed 31 Oct. 2025.

[4]**World Bank.** "Tertiary graduates from Ag, Vet, or Forestry programs (% of population)" *World Development Indicators*, The World Bank Group, https://data.worldbank.org/indicator/SE.TER.GRAD.AG.ZS. Accessed 15 Aug. 2025.

[5]**World Bank.** "Tertiary graduates from Healthcare or Welfare programs (% of population)" *World Development Indicators*, The World Bank Group, https://data.worldbank.org/indicator/SE.TER.GRAD.AG.ZS. Accessed 15 Aug. 2025.
[6]**World Bank.** "Literacy rate, adult total (% of people ages 15 and above)" *World Development Indicators*, UN Educational, Scientific and Cultural Organization (UNESCO), https://data.worldbank.org/indicator/SE.ADT.LITR.ZS?view=chart. Accessed 31 Oct. 2025.
[7]**World Bank.** "Government expenditure on education, total (% of government expenditure)"*World Development Indicators*, UN Educational, Scientific and Cultural Organization (UNESCO), https://data.worldbank.org/indicator/SE.XPD.TOTL.GB.ZS. Accessed 31 Oct. 2025.
[8]**World Bank.** "Current health expenditure (% of GDP)" *World Development Indicators*, UN Educational, Scientific and Cultural Organization (UNESCO), https://data.worldbank.org/indicator/SH.XPD.CHEX.GD.ZS. Accessed 31 Oct. 2025.
[9]**World Bank.** "Gini index" *World Development Indicators*, UN Educational, Scientific and Cultural Organization (UNESCO), https://data.worldbank.org/indicator/SI.POV.GINI. Accessed 31 Oct. 2025.
[10]**World Bank.** "Poverty headcount ratio at national poverty lines (% of population)" *World Development Indicators*, The World Bank Group, https://data.worldbank.org/indicator/SI.POV.GINI. Accessed 31 Oct. 2025.
[11]**World Bank.** "Access to electricity (% of population)" *World Development Indicators*, The World Bank Group, https://data.worldbank.org/indicator/SI.POV.GINI. Accessed 31 Oct. 2025.
[12]**World Bank.** "Land area (sq. km)" *World Development Indicators*, Food and Agriculture Organization of the United Nations (FAO), https://data.worldbank.org/indicator/AG.LND.TOTL.K2. Accessed 31 Oct. 2025.
[13]**World Bank.** "Urban population (% of total population)" *World Development Indicators*, World Urbanization Prospects United Nation (UN), https://data.worldbank.org/indicator/SP.URB.TOTL.IN.ZS. Accessed 31 Oct. 2025.
[14]**World Bank.** "Employment in agriculture (% of total employment) (modeled ILO estimate)" *World Development Indicators*, ILO Modelled Estimates database (IOLEST) International Labour Organization (ILO), https://data.worldbank.org/indicator/SL.AGR.EMPL.ZS. Accessed 31 Oct. 2025.
[15]**World Bank.** "NTD interventions, people requiring interentions against NTDS (number)" *World Health Organization,* The Global Health Observatory, https://www.who.int/data/gho/data/indicators/indicator-details/GHO/reported-number-of-people-requiring-interventions-against-ntds#:~:text=In%202022%2C%201.62%20billion%20people,fewer%20than%20reported%20in%202021. Accessed 31 Oct. 2025.
[16]**SEDAC.** "Gridded Population of the World (GPW) v4" *Earthdata*, The Socioeconomic Data and Applications Center, https://www.earthdata.nasa.gov/data/projects/gpw. Accessed 15 Aug. 2025.
[17]**GRDI.** "Global Gridded Relative Deprivation Index (GRDI) Subnational Human Development Index (SHDI)" *Earthdata*, The Socioeconomic Data and Applications Center, https://www.earthdata.nasa.gov/data/catalog/sedac-ciesin-sedac-pmp-grdi-2010-2020-1.00. Accessed 15 Aug. 2025.
[18]**Gilbert et al. 2022.** "Global cattle distribution in 2015" *Food and Agriculture Organization*, Gridded Livestock of the World (GLW 4),

https://dataverse.harvard.edu/dataset.xhtml?persistentId=doi:10.7910/DVN/LHBICE. Accessed 15 Aug. 2025.
[19]**Tuanmu et al. 2015.** "Dissimilarity" *EarthEnv*, Global Habitat Heterogeneity, https://www.earthenv.org/texture. Accessed 15 Aug. 2025.
[20]**Olson et al. 2004.** "Terrestrial Ecoregions of the World" *World Wildlife Fund - US*, Conservation Science Program, https://www.earthenv.org/texture. Accessed 15 Aug. 2025.
[21]**Rubel et al. 2017.** "Köppen-Geiger Climate Classification" *Vetmed Uni Vienna*, Rubel and Kottek 2010 World Maps of Köppen-Geiger Climate Classification, https://koeppen-geiger.vu-wien.ac.at/. Accessed 15 Aug. 2025.
[22]**ACLED.** "Total conflicts" *ACLED Conflict Exposure,* Raleigh et al 2023 Assessing and Mapping Global and Local Conflict Exposure, https://www.earthenv.org/texture. Accessed 15 Aug. 2025.
[23]**ACLED.** "Percentage of the population exposed to conflict" *ACLED Conflict Exposure,* Raleigh et al 2023 Assessing and Mapping Global and Local Conflict Exposure, https://www.earthenv.org/texture. Accessed 15 Aug. 2025.
[24]**IVSA.** "IVSA chapter presence" *International Veterinary Student Association*, Membership Directory, https://www.ivsa.org/membership-directory/corporate. Accessed 15 Aug. 2025.
